# Current Challenges and Advances on Infectious Diseases in Solid Organ Transplantation

**DOI:** 10.3389/ti.2024.13856

**Published:** 2024-10-09

**Authors:** Mario Fernández-Ruiz, Maddalena Giannella, Ilkka Helanterä, Oriol Manuel, Ligia Camera Pierrotti, Dafna Yahav

**Affiliations:** ^1^ Unit of Infectious Diseases, Hospital Universitario “12 de Octubre”, Instituto de Investigación Hospital “12 de Octubre” (imas12), Centro de Investigación Biomédica en Red de Enfermedades Infecciosas (CIBERINFEC), Instituto de Salud Carlos III, School of Medicine, Universidad Complutense, Madrid, Spain; ^2^ Department of Medical and Surgical Sciences, Bologna and Infectious Diseases Unit, IRCCS Azienda Ospedaliero-Universitaria di Bologna, Alma Mater Studiorum University of Bologna, Bologna, Italy; ^3^ Transplantation and Liver Surgery, Helsinki University Hospital, Helsinki, Finland; ^4^ Infectious Diseases Service and Transplantation Center, Lausanne University Hospital and University of Lausanne, Lausanne, Switzerland; ^5^ Infectious Diseases Division, Hospital das Clinicas, Universidade de São Paulo, São Paulo, Brazil; ^6^ Infectious Diseases Unit, Sheba Medical Center, Ramat Gan, Israel; ^7^ School of Medicine, Faculty of Medical and Health Sciences, Tel Aviv University, Tel Aviv-Yafo, Israel

**Keywords:** cytomegalovirus, multidrug resistant bacterial infection, pancreas allograft, oncogenic viruses, antifungals

Infection remains one of the most common complications after organ transplantation. The epidemiology of infection in solid-organ transplant (SOT) recipients is shaped by the interplay of two key factors: the lifelong use of immunosuppressive drugs impairing cellular immunity, and the surgical procedure itself, along with the subsequent hospital stay [1]. SOT recipients are prone to develop a wide range of infections, caused by opportunistic pathogens like cytomegalovirus (CMV) and molds, to more common healthcare-associated infections, which may sometimes be caused by multidrug-resistant (MDR) organisms. Additionally, certain pathogens can be linked to oncogenic processes triggered by a loss of immune control.

Transplant infectious diseases cover therefore a broad spectrum of research areas, including viral immunology, infection control strategies for MDR organisms, and complications related to immunosuppression, among others. This diversity in managing infections in SOT recipients is highlighted in this Special Issue titled “*Current Challenges and Advances in Infectious Diseases in Solid Organ Transplantation*.”

Serris et al. summarized the 2023 Transplantation and Infection group annual meeting. Topics discussed included antibiotic and non-antibiotic approaches to manage various infections in SOT recipients. Innovative strategies to protect the gut microbiome are still under research, including fecal transplantation and new molecules inactivating non-absorbed antibiotics in the gastrointestinal tract. New antibiotic and antifungal drugs and the evidence to support their use in SOT recipients were reviewed. Gaps in knowledge regarding management of asymptomatic bacteriuria after kidney transplantation (KT) were discussed, including recent evidence to support avoiding antibiotic treatment in the first 2 months following transplantation. Type and duration of therapy for pyelonephritis, as well as innovative approaches for therapy and prevention are also discussed (Serris et al.)

Matuschik et al. reported the results of 138 ABO-incompatible KT procedures performed at Freiburg Transplant Center from 2004 to 2020. This retrospective study compared the use of single-use antigen-selective ABO columns (81 patients) versus reusable nonantigen-specific immunoglobulin adsorption columns (57 patients) and found that use of the latter was associated with 3-fold increased risk for severe and recurrent post-transplant viral and bacterial infections, mainly urosepsis. Rates of allograft rejection were significantly higher with antigen selective ABO columns (29% vs. 14%), though graft survival was similar. Two years mortality was significantly higher with non-antigen specific immunoadsorption (Matuschik et al.)

Walti et al. comprehensively reviewed the latest advancements in the management of refractory and/or resistant CMV infection (R/R CMV) and disease. As highlighted by the authors, R/R CMV constitutes a challenging complication associated to worse graft and patient outcomes, which is in part explained by the common occurrence of drug toxicities with the use of options available to date (i.e., foscarnet or cidofovir). The results of phase 2/3 clinical trials with maribavir and letermovir are critically discussed, as well as controversial questions regarding the risk of emerging resistance, the benefit expected from combination therapy or secondary prophylaxis, or the optimal donor source for CMV-specific T-cells for adoptive immunotherapy. Observational studies exploring the potential role of letermovir for the treatment of R/R CMV were also scrutinized. Finally, the review offers a valuable summary of authors’ institutional guidelines and their personal view on this topic (Walti et al.)

The overall clinical picture of other herpesviruses relevant to the SOT population due to their oncogenic potential—Epstein-Barr virus (EBV) and human herpesvirus 8 (HHV8)— was covered by (Atamna et al.). The authors provided a thorough, albeit concise, overview of the epidemiology, risk factors, diagnosis, and state-of-the-art therapeutic approaches for post-transplant HHV8 disorders (Kaposi´s sarcoma, multicentric Castleman disease, primary effusion lymphoma and inflammatory cytokine syndrome) and EBV-related post-transplant lymphoproliferative disease. Unmet needs in the management of these complications, such as the optimal screening strategy for HHV8 and EBV DNAemia or the pre-emptive use of antivirals or rituximab in case of persistent and/or high-level replication, were also discussed (Atamna et al.)

Namsiripongpun et al. reported a prospective study of 81 KT recipients in Thailand who were monitored with a non-specific interferon (IFN)-γ ELISpot assay at transplantation and at 1 month. The main outcome of interest was CMV infection. In multivariable models, low IFN-γ ELISpot response at 1 month was an independent predictor of CMV infection. Of the patients with low IFN-γ response, >60% developed CMV infection compared to 20% among patients with higher response. This study, together with previous published literature, supports the concept that the risk of later CMV infection can be predicted by also non-specific cellular immune responses (Namsiripongpun et al.)

The review by Bestard et al. gives a comprehensive overview of immunobiology of CMV in transplantation and reviews the current evidence for assessing CMV-specific cell-mediated immunity (CMV-CMI). The potential of CMV-CMI assays to predict the risk of infection has been well described, but until recently clinicians have lacked data and advice on how to implement these assays to aid decision-making in clinical practice. The review very elegantly highlights the literature on both observational and interventional trials and gives practical recommendations and future directions on how to optimize the clinical use of the CMV-CMI assays (Bestard et al.)

The review by Lombardi et al. provides a complete dissection of the antibiotics active against MDR Gram-negative bacteria approved over the last years, specifically ceftolozane/tazobactam, ceftazidime/avibactam, meropenem/vaborbactam, imipenem/relebactam, cefiderocol and eravacycline. Activity spectrum, toxicity profile, clinical use, and PK/PD properties including therapeutic drug monitoring in the setting of liver transplantation were reviewed for each agent. The authors underlined the need of studies on the safety and optimal employment of these drugs in liver transplant recipients.

SOT recipients are particularly vulnerable to MDR organisms, which significantly contribute to morbidity and mortality. Freire et al. addresses the gap in systematic reporting of MDR organism prevalence, especially across high-income (HIC) and low- and middle-income countries (LMIC), where diagnostic tools, screening practices, and drug availability vary. The review focuses on major MDR Gram-negative organisms like Enterobacterales, *Pseudomonas aeruginosa*, and *Acinetobacter baumannii*. It highlights the need for advanced diagnostics and access to new antibiotics to improve outcomes in SOT recipients. Standardization in MDR organism reporting and global epidemiological understanding remains a critical challenge.

Grasberger et al. performed a retrospective study in Finland aimed at assessing the total burden of infections in recipients of simultaneous pancreas-kidney transplantation (SPK) compared with kidney transplantation alone (KTA). The authors compared infection-related hospitalizations and bacteremias during 1- and 5-year follow-up after transplantation, among 162 SPK and 153 type 1 diabetics KTA patients. The inclusion criteria of donor and recipient were age <60 and BMI <30. During the first year, SPK patients had more infection-related hospitalizations (0.54 vs. 0.31 PPY, IRR 1.76, p < 0.001) and bacteremias (0.11 vs. 0.01 PPY, IRR 17.12, p < 0.001) compared to KTA patients. SPK was an independent risk factor for infection-related hospitalization and bacteremia during the first-year post-transplant, but not during the 5-year follow-up. Patient survival did not differ between groups, however, KTA patients had inferior kidney graft survival.

SOT recipients are at an elevated risk for invasive mold diseases (IMD). Isavuconazole, a novel broad-spectrum antifungal agent, has shown a favorable profile, with good tissue penetration, minimal drug interactions, and fewer adverse effects compared to other azoles like voriconazole and posaconazole. Silva et al. conducted an extensive literature review on isavuconazole use in IMD treatment for SOT recipients. The review included 145 SOT patients, mostly lung and kidney transplant recipients, treated with isavuconazole mainly for *Aspergillus* infections. The drug was well-tolerated, with manageable drug-drug interactions with immunosuppressive agents. The authors have concluded that isavuconazole presents as a viable alternative for IMD treatment in this population, warranting further prospective studies.

In conclusion, this Special Issue provides a comprehensive overview of the epidemiology, prevention, and treatment of a wide range of transplant infectious diseases. It emphasizes the importance of novel multidisciplinary management strategies to enhance allograft and patient outcomes.
